# Vertebral Artery Dissection Following Anterior Cervical Decompression and Artificial Disc Replacement

**DOI:** 10.3390/diagnostics13040595

**Published:** 2023-02-06

**Authors:** Seong Hwan Ahn, Haksung Kim, Dae Kyun Kim, Seok Won Kim

**Affiliations:** 1 Department of Neurology, College of Medicine, Chosun University, Gwangju 61453, Republic of Korea; 2 Department of Neurosurgery, College of Medicine, Chosun University, Gwangju 61453, Republic of Korea

**Keywords:** vertebral artery, dissection, anterior cervical approach

## Abstract

Vertebral artery dissection (VAD) is a rare vascular cause of acute stroke. Although VAD may be classified as spontaneous or traumatic, it is increasingly recognized that trivial mechanical stress typically precipitates this potentially dangerous condition. Herein, we report a rare case of VAD and acute stroke following anterior cervical decompression and artificial disc replacement (ADR). To our knowledge, there have been no other cases of acute vertebrobasilar stroke caused by VAD following anterior cervical decompression and ADR. This case highlights that, although rare, acute vertebrobasilar stroke may occur after the anterior cervical approach.

## Figures and Tables

**Figure 1:**
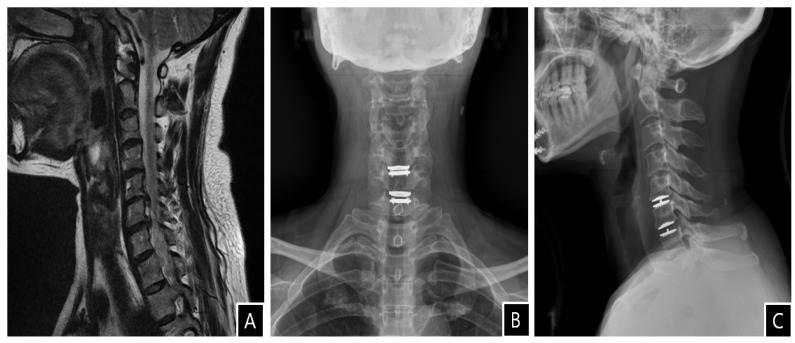
(**A**) T2-weighted sagittal magnetic resonance image reveals foraminal stenosis at the C5–C6 and C6–C7 levels. (**B**,**C**) Postoperative simple radiographs show cervical disc arthroplasty implants at the C5–C6 and C6–C7 levels. A 51-year-old woman with a medical history of hypertension presented with a 6-month history of progressive radiculopathy of the left side that had not responded to conservative treatment. Magnetic resonance imaging (MRI) revealed foraminal stenosis and focal cord compression at the C5–C6 and C6–C7 levels (Figure 1A). She was offered and consented to surgery in the form of anterior discectomy and ADR. The routine laboratory test results of the blood and electrolytes were within the normal ranges. Anesthetic preoperative assessment 2 weeks prior to surgery also showed no specific findings. She underwent elective C5–C6 and C6–C7 anterior cervical decompression and ADR using Mobi-C prostheses (LDR Spine, Zimmer) (Figure 1B,C).

**Figure 2:**
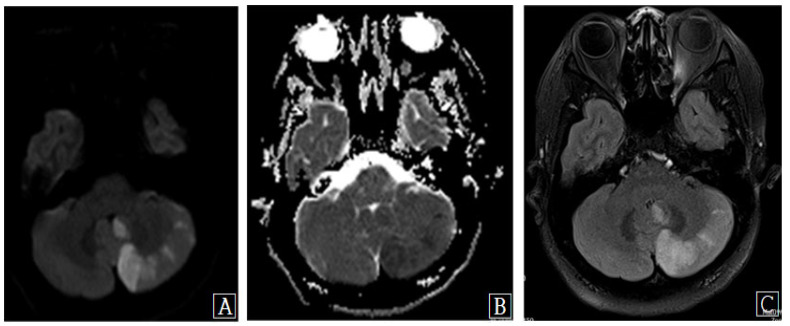
Brain magnetic resonance (MR) images show multiple patchy foci of diffusion restriction, consistent with acute left posterior inferior cerebellar artery territory infarction. (**A**) Diffusion-weighted MR image. (**B**) Apparent diffusion coefficient MR image. (**C**) Axial T2 flair MR image. Uneventful surgery was performed in the usual manner using a right-sided retropharyngeal approach. General anesthesia was uncomplicated, and intraoperative bleeding was well controlled, with a total blood loss of <140 mL. During the surgical procedure, the blood pressure was stable, remaining between 90 and 130 mmHg. No episodes of excessive flexion, extension, or lateral rotation occurred at any stage of the procedure. She awoke from the anesthetic neurologically intact; however, 14 h later, she complained of severe headache, dizziness, and diplopia. Urgent MRI of the cervical spine and brain was performed. MRI of the cervical spine showed satisfactory decompression of the cord and foramen; however, a large left inferior cerebellar signal abnormality indicating an acute cerebellar infarction was found on brain MRI (Figure 2).

**Figure 3:**
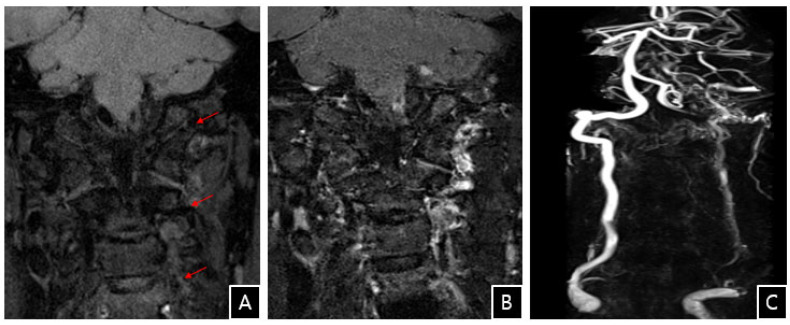
(**A**–**C**) Coronal T1-weighted and enhanced MR and MR angiography images demonstrate occlusion of vertebral artery with eccentric wall thickening in the left distal V2, V3, and V4 segments and strong enhancement with mural thrombus in those segments (arrows). Magnetic resonance angiography demonstrated occlusion of the left VA and dissection of the left V2–V4 segments (Figure 3). Extraluminal pathology compressing the left distal VA was also noted. Thrombolysis was not recommended after the surgery, and a stroke consultant commenced aspirin therapy. She was transferred to the stroke unit and underwent stroke rehabilitation with occupational therapy support. At the 6-month follow-up, she had no residual headache, dizziness, imbalance, or diplopia. Most reported cases of VA complications related to cervical spine surgery are a result of direct operative injury, compression, or traction of the VA or far-lateral bone drilling [1,2,3]. There are a few cases of VADs in which no direct injury has been reported. The exact pathophysiology of this uncommon complication is difficult to determine given that there is no evidence of abrupt trauma to the VA during surgery due to direct injury, forced neck rotation, stretching, or hyperextension. Spontaneous dissection of the VA has been reported in the setting of genetic changes, such as low levels of alpha-1 antitrypsin, various genetic polymorphisms, hyperhomocysteinemia, gene mutations, the presence of migraines, and vessel abnormalities. Environmental factors, including oral contraceptive use and infectious conditions, have also been reported [4]. Dickerman et al. reported that VAD resulted in posterior circulatory stroke after anterior cervical corpectomy. In the absence of direct injury to blood vessels, they hypothesized that traction may be implicated [5]. Sheth et al. also reported delayed VAD following uncomplicated posterior cervical foraminotomy without direct injury. They postulated that vibrational shear stress through the bone during surgery contributed to vessel wall injury and subsequent thrombosis [6]. It has been reported that vibration between 60 and 800 Hz in the arteries of rats increased discontinuities in the internal elastic membrane with patches of missing tunica intima. Moreover, endotracheal intubation may have caused VAD. Hyperextension of the neck during intubation or intraoperatively may lead to stretching and injury of cervical vessels and cause dissection of brain-supplying arteries [7]. Mechanical impingement by hyperextension or neck rotation can cause profound vertebral artery hypoperfusion leading to occlusion. Alternatively, cerebral hemodynamic changes during sustained hyperextension of the neck have been previously noted in patients with hypoplasia of the neck vessels and vertebral occlusion, stenosis, or prior ischemic disease. Our patient had none of these risk factors for spontaneous VAD, and dissection occurred at the surgical site. It was difficult to attribute the injury to anything other than indirect or direct perioperative injuries. Despite the various possibilities of the development of VAD, the precise cause of VAD in our patient remains unexplained. Cervical ADR is a more complicated procedure than anterior cervical discectomy and fusion (ACDF) and it should be implanted more carefully. If an artificial disc device is the wrong size or incorrectly implanted, it can cause more complications, such as continuing pain or the need for revision surgery. In this particular case, although extremely rare, acute vertebrobasilar stroke caused by VAD without risk factors or direct injury is a potentially serious complication following anterior cervical decompression and ADR.

## Data Availability

Not applicable.
